# Heat-Stroke

**Published:** 1888-08-18

**Authors:** 


### THE DOCTOR'S PULPIT.

### HEAT-STROKE.

HEAT-STROKE may be taken as almost synonymous with sunstroke. It does not seem to be generally understood that in very hot weather alarming and dangerous consequences may result from heat alone, apart from exposure to the rays of the sun. This year of grace, 1888, will long be remembered as a year without spring and with an amputated summer. Captain Cuttle had lost an arm, but it was wonderful what he could do with his hook. The present summer, though "docked," as "horsey" people say, of its length, may yet work marvels with what remains of its days and weeks. The sudden onslaught of hot weather which commenced on Wednesday week was highly dangerous on account both of its suddenness and its intensity. Usually, even in our climate, there is a little preliminary sunshine in the late spring, and a little more in the early summer, which gives us at least a modicum of training in the endurance of heat. This year there has been no such period of preparation. After being enveloped in raw winds and rains resembling those of an ordinary November, we were plunged all at once and without warning into a limitless bath of what some bold and original advertiser has called "liquid sunshine."

It does not seem to be generally understood that the British people of this generation are more liable to injury from excessive heat than their fathers and grandfathers were. The reason for this is not to be sought in the regions of meteorology, but in the more practical domains of everyday experience. The habits of the people have changed. Those who formerly worked a little with their brains now work much, and those who then used their brains not at all are now compelled to use them a little. We do not affirm that the brains of this generation are better than those of the last two or three, but only that in certain directions they are more generally used. But in heat or sunstroke it is practically always the brain that suffers. The working brain is the susceptible brain: susceptible in every direction, and therefore, of course susceptible to heat. A man with thick bushy hair and a very dense and solid skull may endure a midday sun in an August harvest-field for hours without risk; whilst a studious clergyman or a city clerk would probably faint with the heat in an hour. Men, women, and children in this age, and particularly those brought up in large towns, are markedly susceptible to the solar heat.

Such being the case, what is the course to pursue? Would it be advantageous to detail the symptoms of sunstroke, and then to expound the treatment? Certainly not! Preventive measures are here entirely efficacious, and are all that any reasonable man or woman requires. It is in such domains of physiology as these that instruction for the people is of unquestionable interest and utility. Once the sunstroke has taken place, self-management is not available. The doctor must be called in. Prevention is the thing to be aimed at, and prevention is within the reach of almost everyone.

All the world knows that the midday sun is most to be dreaded. At that period the rays are shortest, and what is of still more importance, they are vertical, beating down directly upon the top of the head. By way of counteraction several things may be done. The first, and obviously the best is to remain indoors for an hour or two at midday if possible. This is so plainly a matter of common sense that even military officers sometimes carry it out in hot climates, although there are not wanting cases in which their invincible devotion to "pipeclay" has caused them to march their men over sandy deserts under a vertical sun, and lead them not to victory but to death at the same time.

The next best protection is a large and stout umbrella. Ladies are to the forefront here with their parasols. Perhaps they are not so much afraid of sunstroke as of their complexions. Be their reason what it may, the parasol is an excellent institution. But what will the male portion of the population say about the umbrella as a protection against the sun's rays? Many of them will say what the learned world said of Moses' paradoxes—nothing at all. But in the name of common s nse, why should a man use an umbrella in a shower of rain and object to use it under a blazing sun? The real fact is that our Englishman is ashamed of being thought a "molly-coddle." But why then is he not ashamed of protecting himself against the rain? We suspect he does not use his umbrella so much for protecting *himself* against the rain as for protecting his *clothes*. It must be confessed that a citizen in a top-hat soaked through and through, and with clothes hanging all limp and wet about him, is a decidedly ridiculous sight. Nevertheless, for really serious consequences a burning sun is much more to be dreaded than a shower of rain, and it becomes full-grown men to weigh and measure with scales of sense and experience. Umbrellas, therefore, and big ones, are the best preventives against sunstroke, next to remaining indoors.

After umbrellas, hats! The tropical "helmet" fulfils most of the conditions of an efficient head-protector. But of course, being "tropical" and a helmet, the average Londoner would be afraid to be seen with it in Pall Mall or the City. What an infinite baby the average Londoner is in many respects. We have no hope that he will be persuaded to try the helmet, but he may at least allow himself to be seen in a "white hat." The perfection of a white hat is that it be light and well ventilated.

Last, but not least, is the cold bath. Not merely a cold bath on rising in the morning, but a cold bath at midday and a cold bath at night, if necessary. To sluice the head well with cold water two or three times a day in hot weather is not only an effective protection against sunstroke, but a tonic of the most invigorating character. Many a man whose life has been endangered by heat might have saved himself from all risk if, at the first feeling of oppression, he had gone to the nearest stream, or tap, and sluiced his head. In London and other large towns, every restaurant should have taps always ready for this purpose.

The conclusion of the whole matter is this: that in really hot weather there is a good deal of danger both in the sun and in the shade; that this danger is not peculiar to old or apoplectic people, but is shared by all—men, women, and children; that it is within the power of almost all to avert the danger entirely by remaining indoors or using umbrellas; that if umbrellas are not tolerated, hats may be substituted, and if hats cannot be procured, a very effective means of keeping the temperature of the head and of the body at a safe level may be found in the nearest stream, at the village pump, or at the water company's tap. These methods of prevention are simple and inexpensive, and well within the reach of almost all.

